# Metabolomics implicate eicosanoids in severe functional mitral regurgitation

**DOI:** 10.1002/ehf2.14160

**Published:** 2022-10-10

**Authors:** Thomas M. Hofbauer, Klaus Distelmaier, Besnik Muqaku, Georg Spinka, Veronika Seidl, Henrike T. Arfsten, Gerhard Hagn, Samuel Meier‐Menches, Philipp E. Bartko, Noemi Pavo, Matthias Hoke, Suriya Prausmueller, Gregor Heitzinger, Dietmar Pils, Irene M. Lang, Christian Hengstenberg, Martin P. Hülsmann, Christopher Gerner, Georg Goliasch

**Affiliations:** ^1^ Department of Cardiology, Internal Medicine II Medical University of Vienna Waehringer Guertel 18‐20 A‐1090 Vienna Austria; ^2^ Herz Zentrum Waehring Vienna Austria; ^3^ Institute of Analytical Chemistry University of Vienna Vienna Austria; ^4^ Joint Metabolome Facility University of Vienna and Medical University of Vienna Vienna Austria; ^5^ Department of Angiology, Internal Medicine II Medical University of Vienna Vienna Austria

**Keywords:** Functional mitral regurgitation, Heart failure, Metabolomics, Eicosanoids

## Abstract

**Aims:**

Secondary, or functional, mitral regurgitation (FMR) was recently recognized as a separate clinical entity, complicating heart failure with reduced ejection fraction (HFrEF) and entailing particularly poor outcome. Currently, there is a lack of targeted therapies for FMR due to the fact that pathomechanisms leading to FMR progression are incompletely understood. In this study, we sought to perform metabolomic profiling of HFrEF patients with severe FMR, comparing results to patients with no or mild FMR.

**Methods and results:**

Targeted plasma metabolomics and untargeted eicosanoid analyses were performed in samples drawn from HFrEF patients (*n* = 80) on optimal guideline‐directed medical therapy. Specifically, 17 eicosanoids and 188 metabolites were analysed. Forty‐seven patients (58.8%) had severe FMR, and 33 patients (41.2%) had no or non‐severe FMR. Comparison of eicosanoid levels between groups, accounting for age, body mass index, and sex, revealed significant up‐regulation of six eicosanoids (11,12‐EET, 13(R)‐HODE, 12(S)‐HETE, 8,9‐DiHETrE, metPGJ2, and 20‐HDoHE) in severe FMR patients. Metabolites did not differ significantly. In patients with severe FMR, but not in those without severe FMR, levels of 8,9‐DiHETrE above a cut‐off specified by receiver‐operating characteristic analysis independently predicted all‐cause mortality after a median follow‐up of 43 [interquartile range 38, 48] months [hazard ratio 12.488 (95% confidence interval 3.835–40.666), *P* < 0.0001].

**Conclusions:**

We report the up‐regulation of various eicosanoids in patients with severe FMR, with 8,9‐DiHETrE appearing to predict mortality. Our observations may serve as a nucleus for further investigations into the causes and consequences of metabolic derangements in this important valvular abnormality.

## Introduction

Secondary, or functional, mitral regurgitation (FMR) is prevalent among patients with heart failure with reduced ejection fraction (HFrEF). FMR is characterized by left ventricular (LV) remodelling and subsequent papillary muscle displacement, resulting in mitral valve (MV) leaflet tethering, dilatation, flattening of the mitral annulus, and reduced closing forces.^1^ FMR is either of ischaemic or, less frequently, non‐ischaemic origin. It is presumed that volume overload on a primary failing ventricle increases diastolic wall stress^2^ and consequently stimulates additional maladaptive processes, leading to further ventricular dilatation and failure.^3^

The natural course of FMR remains unclear. Approximately 20% of HFrEF patients with non‐severe FMR developed severe regurgitation over a period of 3 years^4^; this was not modulated by ischaemic vs. non‐ischaemic origin of mitral regurgitation (MR).

Even when treated with guideline‐directed medical therapy (GDMT) recommended by established European guidelines for HF,^5^ prognosis in patients with severe FMR remains unacceptably poor. Even GDMT is often insufficient to prevent FMR deterioration.^4^ In one study, cardiac mortality after 4 years was 6% for mild, 43% for moderate, and 45% for severe FMR.^6^ Extending this follow‐up period, we recently showed that FMR confers poor outcome, with a hazard ratio (HR) of 1.76 for mortality after a follow‐up of 8 years.^7^ Intriguingly, FMR affected outcome only in patients with moderate LV dysfunction, indicating that with HF progressing to a state beyond repair, the therapeutic opportunity for MV intervention is closed.^7^

Thus, it is of increasing interest to further characterize the role of FMR in HFrEF.^8^ Owing to technical progress, it is increasingly possible to biologically assess these patients in even greater detail. Such advances offer new insight into the role of FMR in HFrEF, as was recently demonstrated by employing metabolomics^9^, ^10^ and eicosanoid analysis.^11^ Eicosanoids, which are involved in a plethora of physiologic mechanisms,^12^ were demonstrated to directly affect cell functions at extremely low concentration ranges.^13^ Importantly, metabolic changes often precede cardiac structural changes in the failing heart, offering the possibility of elucidating the pathophysiology of FMR in these patients, closing the conceptual gap between cardiac metabolism, aberrant signalling, and functional valve lesions in HFrEF,^14^, ^15^ and providing information on biomarkers of disease complementary to imaging techniques.^16^

This study aimed to profile a pool of targeted plasma metabolites in addition to eicosanoids obtained from HFrEF patients with severe FMR to elucidate its pathophysiologic associations with the circulating metabolome.

## Methods

### Study population

In this observational study, patients with chronic HFrEF and either severe FMR or no/mild FMR were enrolled between February 2016 and March 2017 at the HF outpatient clinic of the Vienna General Hospital, a university‐affiliated tertiary centre. HFrEF was defined in line with the current HF guidelines,^5^ that is, symptoms and/or signs of HF and a left ventricular ejection fraction (LVEF) below 40%. MR was quantified by an integrated approach comprising MV morphology, width of the proximal regurgitant jet, and proximal flow convergence.^17^ MV vena contracta width (VCW), MV jet area, and severe FMR were defined as previously described.^7^ Patients with more than mild aortic or mitral stenosis or ≥ moderate primary MR were excluded. The investigation conforms with the principles outlined in the Declaration of Helsinki and was approved by the Ethics Committee of the Medical University of Vienna (Approval Reference Number 1431/2017).

### Clinical and routine laboratory measures

Medical history, current medication, electrocardiogram recording, and a transthoracic echocardiogram were collected at study enrolment. Risk factors were recorded according to the respective guidelines.^18^ According to the standard operating procedure of our HF outpatient clinic and in agreement with the guidelines,^19^ dosage of medical therapy was pro‐actively increased in all enrolled patients until the maximum recommended dosage was reached or a further increase was no longer possible due to clinical characteristics of the patient (systolic blood pressure < 90 mmHg, heart rate < 60 b.p.m., potassium level > 5.0 mmol/L). Venous blood samples were drawn from an antecubital vein. Routine laboratory parameters were analysed according to the local laboratory's standard procedure. For subsequent analyses, samples were centrifuged at 2000 *g* for 10 min, and plasma was stored in aliquots at −80°C.

### Targeted metabolomics and untargeted eicosanoid analysis

Targeted metabolomics experiments were conducted using the AbsoluteIDQ p180 kit (Biocrates Life Sciences AG), enabling the detection and (semi)quantitation of up to 188 analytes, comprising 40 acylcarnitines, 42 amino acids and biogenic amines, 15 sphingolipids, 90 glycerophospholipids, and the sum of hexoses. All samples measured were collected between February 2016 and March 2017. Measurements were carried out in September 2017 using liquid chromatography–mass spectrometry (LC–MS) and flow injection (FIA)‐MS analyses on a 4000 QTRAP MS system (AB Sciex) coupled to a 1200 RR HPLC system (Agilent), utilizing the Analyst 1.6.2 software (also AB SCIEX). All required standards, quality controls, and eluents were included in the kit as well as the chromatographic column for the LC–MS/MS analysis part. Phenyl isothiocyanate (Sigma‐Aldrich) was purchased separately and used for derivatization of amino acids and biogenic amines according to the kit manual. Preparation of the measurement worklist as well as data validation and evaluation was performed with the software supplied with the kit (MetIDQ, Version 5‐4‐8‐DB100‐Boron‐2607, Biocrates Life Sciences).

The untargeted eicosanoid analysis was performed essentially as described previously.^20^ For eicosanoid sample collection and preparation, blood plasma (0.5 mL) was added to cold ethanol (2 mL, abs. 99%, −20°C; AustroAlco) containing an internal standard mixture of 12S‐HETE‐d8, 15S‐HETE‐d8, 5‐Oxo‐ETE‐d7, 11,12‐DiHETrE‐d11, PGE‐d4, and 20‐HETE‐d6 (each 100 nM; Cayman Europe). The samples were stored overnight at −20°C. After centrifugation (30 min, 2000 g, 4°C), the supernatant was transferred to a new 15 mL Falcon™ tube and ethanol was evaporated via vacuum centrifugation (37°C) until the original sample volume was accomplished. Samples were loaded on preconditioned StrataX solid phase extraction columns (30 mg/mL; Phenomenex) using glass Pasteur pipettes. Columns were washed with MS‐grade water (3 mL) and elution of eicosanoids was performed with ice‐cold methanol (500 μL, abs.; VWR International) containing 2% formic acid (Sigma‐Aldrich). Methanol was evaporated under a gentle stream of nitrogen at room temperature and the samples were reconstituted in 150 μL reconstitution buffer (H_2_O : ACN : methanol + 0.2% formic acid − vol% 65:31.5:3.5), including a second mixture of internal standards, for example, 5S‐HETE‐d8, 14,15‐DiHETrE‐d11, and 8‐iso‐PGF2a‐d4 (10–100 nM; Cayman Europe). Reconstituted samples were stored at 4°C and measured subsequently via LC–MS/MS using a QExactive orbitrap HF (ThermoFisher) hyphenated with a Vanquish UHPLC (ThermoFisher). Results of eicosanoids were normalized to each other (Supporting Information, *Figure* *S1*).

### Statistical analysis

Distribution of data was tested using the Kolmogorov–Smirnov test, the Shapiro–Wilk test, and histograms. Data are expressed as means ± standard deviation (SD) in case of normal distribution or as median [interquartile range (IQR)]. To compare normally distributed variables, paired Student's *t*‐test was used. Otherwise, the Wilcoxon signed‐rank test was applied. Comparisons between more than two groups were conducted using one‐way ANOVA. Pearson's or Spearman's rank correlation was applied to calculate correlations, depending on data distribution. Discrete data are given as count and percentage. Receiver‐operating characteristic (ROC) analysis was employed to assess the discriminatory power of experimental variables on outcome. Cut‐offs were defined by maximizing the Youden index, calculated as *Y = sensitivity + specificity − 1*. Mortality was analysed using Cox regression modelling. For multivariable adjustment, only parameters with a *P*‐value < 0.10 in univariable analyses were used. Data are presented as HRs and 95% confidence intervals (CIs).

Raw abundance values of eicosanoids were log_2_ transformed, replacing values of 0 with 0.0001, and normalized over all patients with the cyclic loess (pairwise) method. Raw abundance values of metabolites were log_2_ transformed, replacing values of 0 with 0.0001. Statistical analyses of differences in eicosanoids and metabolites between severe and no/mild FMR were performed in R using R‐package limma v3.42.2.^21^ Concrete linear modelling and an empirical Bayesian approach was used to moderate the standard errors across analytes, that is, shrink towards a common value. Information of age, body mass index (BMI), and sex was included in the models and, therefore, results were corrected for these three possible confounders. Correction for multiple testing was done by the Benjamini–Hochberg method, yielding false discovery rates (FDRs). FDRs < 0.1 were considered as statistically significant.

A two‐sided *P*‐value below 0.05 was considered significant. Statistical analyses were performed using IBM SPSS Statistics 26.0 for Windows. Figures were generated using GraphPad Prism 8. Numerical data are depicted as scatter plots and box plots, with whiskers defined according to Tukey's method. For survival analyses, the Kaplan–Meier curves are provided.

## Results

### Patient characteristics

We enrolled a total of 80 patients with chronic HFrEF. Median age was 64 [51, 73] years; 63 patients (78.8%) were male. Forty‐seven (58.8%) patients had severe FMR; 33 (41.2%) patients had no or mild FMR. Baseline characteristics were similar between the two patient groups, except for hyperlipidaemia, which was less frequent in patients with severe FMR. Overall median N‐terminal pro‐brain natriuretic peptide (NT‐proBNP) was 2032 [772, 5066] pg/mL, with no differences between patient groups. LV function was significantly reduced (i.e. at least moderately reduced) in all patients. Systolic and diastolic blood pressures were 125 [108, 148] and 78 [70, 85] mmHg, respectively. Both systolic and diastolic pressures were lower in severe FMR patients. Transthoracic echocardiography revealed left atrial diameter to be increased in severe FMR. Tricuspid regurgitation was more frequent in severe FMR, whereas systolic pulmonary artery pressure was similar between groups. As expected, MV VCW and jet area were higher in severe FMR.

Seventy‐six (95.0%) patients received beta‐blockers up‐titrated to a median dose of 100% of the maximum guideline‐recommended dosages. For renin angiotensin system (RAS) antagonists, 76 (95.0%) patients had dosages up‐titrated to 100% of the guideline‐recommended maximum. A total of 56 (70.0%) patients were treated with a mineralocorticoid receptor antagonist, whereas 33 (41.3%) patients received furosemide. Twenty‐three (28.7%) patients were prescribed acetylsalicylic acid; 34 (42.5%) were on statin therapy. Between the two patient groups, no differences in medication were observed, except for statins, which were prescribed more frequently in the no/mild FMR group. Lipid levels between patient groups did not differ significantly.

Detailed baseline characteristics according to FMR severity are displayed in *Table* *1*.

**Table 1:** Baseline characteristics of the study population (*n* = 80)

Baseline characteristics	All patients (*n* = 80)	No/mild FMR (*n* = 33)	Severe FMR (*n* = 47)	*P*‐value
Age, years, median [IQR]	64 [51, 73]	63 [49, 71]	66 [53, 74]	0.302
Male sex, *n* (%)	63 (78.8)	28 (84.8)	35 (74.5)	0.406
BMI, kg/m^2^, median [IQR]	26.1 [23.1, 30.8]	27.3 [24.1, 33.2]	25.3 [22.3, 30.0]	0.060
Ischaemic aetiology of HF, *n* (%)	29 (36.3)	15 (45.5)	14 (29.8)	0.165
Arterial hypertension, *n* (%)	52 (65.0)	24 (72.7)	28 (59.6)	0.245
Diabetes mellitus, *n* (%)	24 (30.0)	13 (39.4)	11 (23.4)	0.143
Hyperlipidaemia, *n* (%)	40 (50.0)	23 (69.7)	17 (36.2)	0.006*
Atrial fibrillation, *n* (%)	30 (37.5)	12 (36.4)	18 (38.3)	1.000
NYHA functional class				0.262
NYHA I, *n* (%)	17 (21.3)	6 (18.2)	11 (23.4)	
NYHA II, *n* (%)	45 (56.3)	22 (66.7)	23 (48.9)	
NYHA III, *n* (%)	18 (22.5)	5 (15.2)	13 (27.7)	
NYHA IV, *n* (%)	0 (0.0)	0 (0.0)	0 (0.0)	
BP_systolic_, mmHg, median [IQR]	125 [108, 148]	135 [116, 158]	120 [100, 130]	0.003*
BP_diastolic_, mmHg, median [IQR]	78 [70, 85]	81 [71, 90]	75 [62, 80]	0.010*
ICD, *n* (%)	32 (40.0)	14 (42.4)	18 (38.3)	0.818
CRT, *n* (%)	14 (17.5)	6 (18.2)	8 (17.0)	1.000
PM, *n* (%)	6 (7.5)	1 (3.0)	5 (10.6)	0.392
**Laboratory measurements**				
Haemoglobin, g/dL, mean ± SD	13.5 ± 1.7	13.8 ± 1.6	13.4 ± 1.7	0.272
Sodium, mmol/L, mean ± SD	139 ± 2.7	140 ± 2.5	139 ± 2.9	0.205
Potassium, mmol/L, mean ± SD	4.9 ± 0.6	4.8 ± 0.6	4.9 ± 0.5	0.250
ASAT, U/L, median [IQR]	24 [21, 30]	25 [22, 32]	24 [21, 28]	0.240
ALAT, U/L, median [IQR]	23 [17, 39]	24 [18, 46]	23 [16, 34]	0.230
gGT, U/L, median [IQR]	42 [23, 95]	43 [22, 97]	42 [23, 93]	0.905
NT‐proBNP, pg/mL, median [IQR]	2032 [772, 5066]	1916 [740, 3521]	2108 [937, 6294]	0.268
CRP, mg/dL, median [IQR]	0.29 [0.13, 0.61]	0.24 [0.14, 0.48]	0.29 [0.11, 0.71]	0.618
TG, mg/dL, median [IQR]	115 [85, 158]	144 [82, 169]	104 [85, 154]	0.197
Total cholesterol, mg/dL, median [IQR]	163 [140, 205]	153 [138, 187]	167 [140, 221]	0.207
HDL‐C, mg/dL, median [IQR]	47 [39, 59]	45 [35, 59]	49 [41, 58]	0.361
LDL‐C, mg/dL, median [IQR]	88 [69, 121]	82 [66, 111]	92 [71, 139]	0.098
Lp(a), nmol/L, median [IQR]	20 [6, 114]	43 [17, 161]	10 [6, 105]	0.147
**Echocardiography**				
LV function reduction				0.074
Moderately (EF 30–40%), *n* (%)	14 (17.5)	9 (27.3)	6 (10.6)	
Severely (EF < 30%), *n* (%)	66 (82.5)	24 (72.7)	42 (89.4)	
LVEDD, mm, mean ± SD	61.3 ± 9.8	59.2 ± 9.8	62.9 ± 9.6	0.094
LA diameter, mm, mean ± SD	65.3 ± 9.9	60.3 ± 7.8	68.9 ± 9.8	<0.0001*
RVEDD, mm, mean ± SD	38.3 ± 7.2	37.1 ± 7.5	39.2 ± 6.9	0.199
TR (≥ moderate), *n* (%)	27 (33.8)	1 (3.0)	26 (55.3)	<0.0001*
sysPAP, mmHg, mean ± SD	50.7 ± 14.3	45.7 ± 12.0	53.1 ± 14.8	0.063
MV VCW, cm, median [IQR]	9.4 [4.0, 12.5]	3.3 [0.1, 5.1]	12.1 [10.0, 14.2]	<0.0001*
MV jet area, cm, median [IQR]	9.6 [4.2, 14.9]	3.2 [0.1, 5.5]	13.7 [10.2, 17.1]	<0.0001*
**Medication**				
Beta‐blockers, *n* (%)	76 (95.0)	31 (93.9)	45 (95.7)	1.000
Recommended dose, %, median [IQR]	100 [50, 100]	75 [50, 100]	100 [50, 100]	
RAS antagonist, *n* (%)	76 (95.0)	32 (97.0)	44 (93.6)	0.639
Recommended dose, %, median [IQR]	100 [50, 100]	50 [50, 100]	100 [50, 100]	
MRA, *n* (%)	56 (70.0)	22 (66.7)	34 (72.3)	0.626
Recommended dose, %, median [IQR]	50 [0, 100]	50 [0, 100]	50 [0, 100]	
Ivabradine, *n* (%)	11 (13.8)	4 (12.1)	7 (14.9)	1.000
SGLT2 inhibitors, *n* (%)	7 (8.8)	4 (12.1)	3 (6.4)	0.439
Furosemide, *n* (%)	33 (41.3)	13 (39.4)	20 (42.6)	0.821
Acetylsalicylic acid, *n* (%)	23 (28.7)	8 (24.2)	15 (31.9)	0.616
P2Y12 inhibitors, *n* (%)	6 (7.5)	4 (12.1)	2 (4.3)	0.224
Statins, *n* (%)	34 (42.5)	19 (57.6)	15 (31.9)	0.038*
Amiodarone, *n* (%)	20 (25.0)	10 (30.3)	10 (21.3)	0.435
Allopurinol, *n* (%)	8 (10.0)	5 (15.2)	3 (6.4)	0.264
Proton pump inhibitor, *n* (%)	38 (47.5)	19 (57.6)	19 (40.4)	0.173

ALAT, alanine transaminase; ASAT, aspartate transaminase; BMI, body mass index; BP, blood pressure; CRP, C‐reactive protein; CRT, cardiac resynchronization therapy; EF, ejection fraction; FMR, functional mitral regurgitation; gGT, gamma‐glutamyl transferase; HDL‐C, high‐density lipoprotein cholesterol; ICD, implantable cardiac defibrillator; IQR, interquartile range; LA, left atrium; LDL‐C, low‐density lipoprotein cholesterol; Lp(a), lipoprotein(a); LV, left ventricular; LVEDD, left ventricular end‐diastolic diameter; MRA, mineralocorticoid receptor antagonist; MV, mitral valve; NT‐proBNP, N‐terminal pro‐brain natriuretic peptide; NYHA, New York Heart Association; PM, pacemaker; RAS, renin angiotensin system; RVEDD, right ventricular end‐diastolic diameter; SD, standard deviation; SGLT2, sodium glucose linked transporter 2; sysPAP, systolic pulmonary artery pressure; TG, triglycerides; TR, tricuspid regurgitation; VCW, vena contracta width.

* *P*‐values < 0.05 were considered statistically significant.

### Eicosanoids are increased in severe functional mitral regurgitation

Using a targeted metabolomic approach, we analysed a total of 188 metabolites. After adjustment for multiple testing, no significant differences were found on a metabolomic level between HFrEF patients with severe FMR and patients without FMR (Supporting Information, *Table* *S1*).

**Figure 1:**
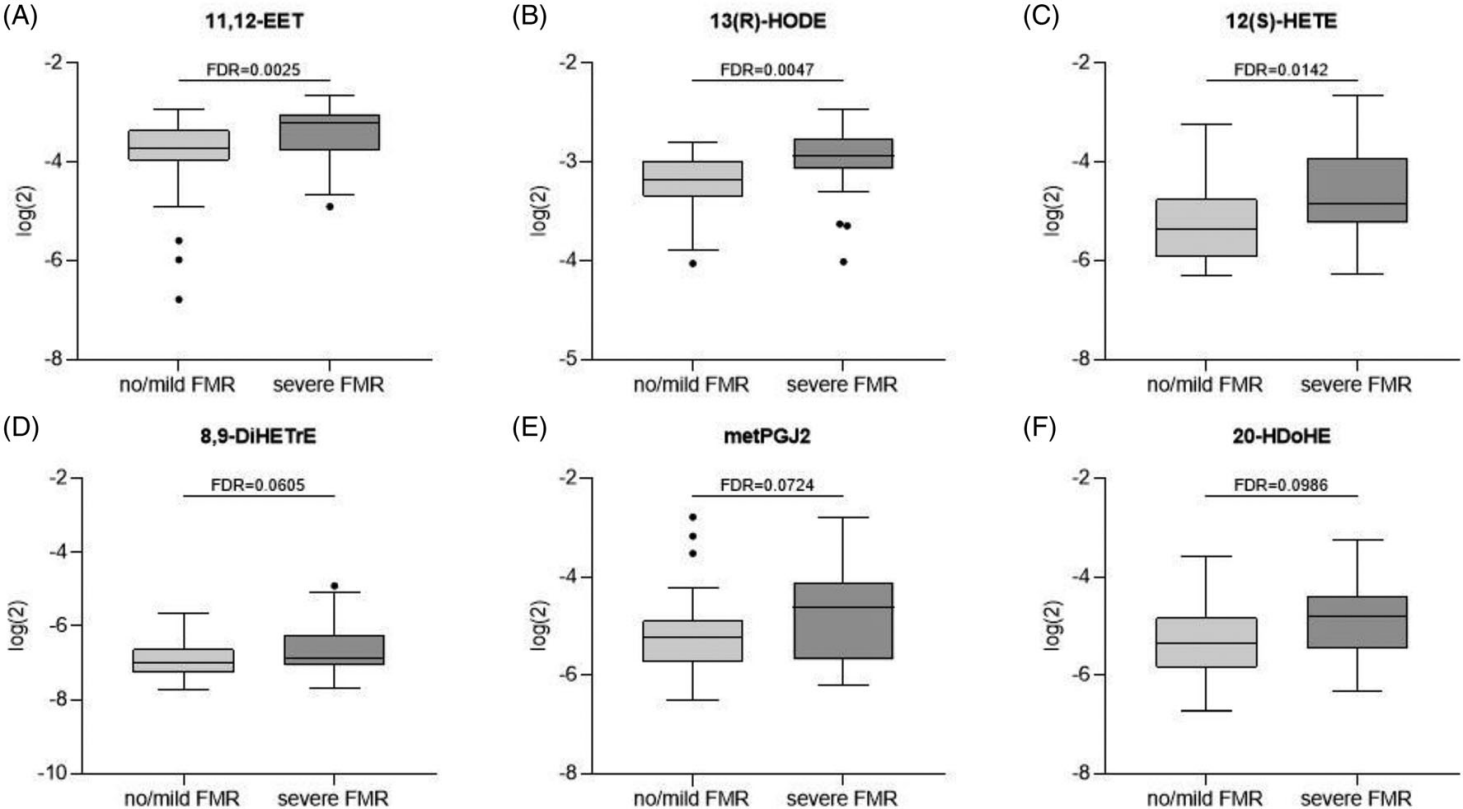
Levels of eicosanoids in patients with no or mild vs. severe functional mitral regurgitation (FMR). Eicosanoids were measured in plasma from patients with or without severe FMR using metabolomic analysis. Out of a total of 17 measured eicosanoids, six were different between the groups, with a false discovery rate (FDR) of below 0.1. (A) 11,12‐EET, (B) 13(R)‐HODE, (C) 12(S)‐HETE, (D) 8,9‐DiHETrE, (E) metPGJ2, and (F) 20‐HDoHE.

**Figure 2:**
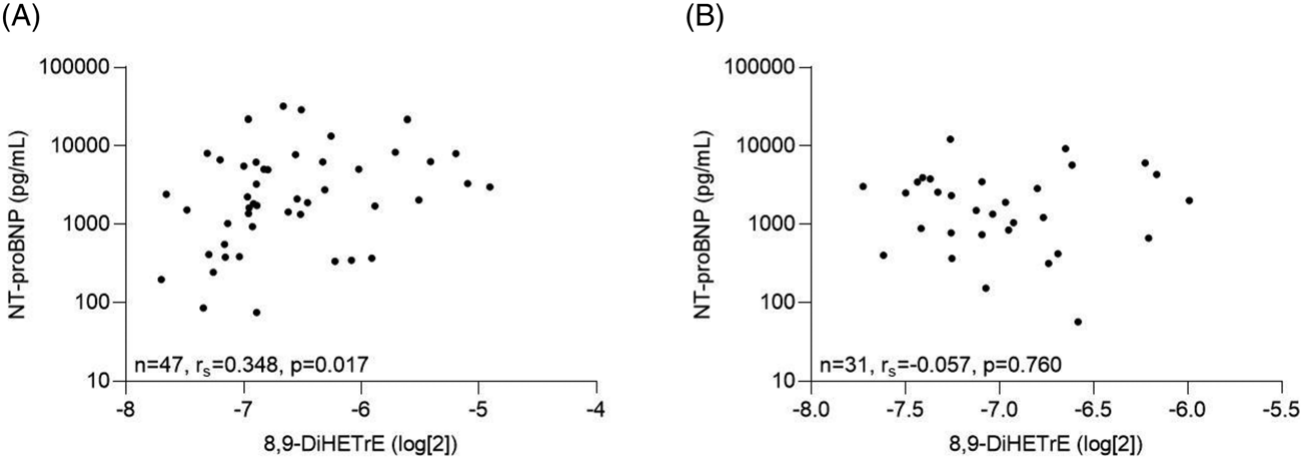
Correlation between eicosanoid 8,9‐DiHETrE and N‐terminal pro‐brain natriuretic peptide (NT‐proBNP) in patients with severe functional mitral regurgitation (FMR). Correlations were calculated using Spearman's coefficient. (A) Severe FMR and (B) no/mild FMR; alpha‐level 0.05.

We next assessed a total of 17 eicosanoids, of which six (11,12‐EET, 13(R)‐HODE, 12(S)‐HETE, 8,9‐DiHETrE, metPGJ2, and 20‐HDoHE) were significantly increased in patients with severe FMR compared with patients with no or non‐significant FMR (*Table* *2*, *Figure* *1*). Information of age, BMI, and sex was considered as confounders and accounted for during statistical testing (Supporting Information, *Figure* *S1*). Neither gender, background medication, nor lipid levels influenced levels of eicosanoids (data not shown). A positive correlation was observed between levels of 8,9‐DiHETrE and NT‐proBNP in patients with severe FMR (*n* = 47, *r*_s_ = 0.348, *P* = 0.017; *Figure* *2* *A*), but not in patients with no/mild FMR (*n* = 31, *r*_s_ = −0.057, *P* = 0.760; *Figure* *2* *B*). Similar correlations were not observed between NT‐proBNP and the other five eicosanoids increased in severe FMR (data not shown). A positive correlation was found between 8,9‐DiHETrE and MV VCW (*n* = 80, *r*_s_ = 0.252, *P* = 0.024). The same was true for 11,12‐EET (*n* = 80, *r*_s_ = 0.341, *P* = 0.002) and 13(R)‐HODE (*n* = 80, *r*_s_ = 0.413, *P* < 0.001). However, upon stratification by FMR severity, these correlations were not observed in either group (data not shown). Tricuspid valve VCW did not correlate with 8,9‐DiHETrE (*n* = 80, *r*_s_ = 0.149, *P* = 0.186) but correlated with 11,12‐EET (*n* = 80, *r*_s_ = 0.308, *P* = 0.005) and 13(R)‐HODE (*n* = 80, *r*_s_ = 0.266, *P* = 0.017).

**Table 2:** Eicosanoids included in targeted metabolomic analysis of patients with severe functional mitral regurgitation

Eicosanoid name	Abbreviation	Log_2_ FC	95% CI	FDR	Figure
11,12‐Epoxyeicosatrienoic acid	11,12‐EET	0.5760	0.2860 to 0.8660	0.0025*	*Figure* *1* *A*
13(R)‐Hydroxy‐9Z,11E‐octadecadienoic acid	13(R)‐HODE	0.2576	0.1143 to 0.4009	0.0047*	*Figure* *1* *B*
12(S)‐Hydroxyeicosatetraenoic acid	12(S)‐HETE	0.6383	0.2297 to 1.0469	0.0142*	*Figure* *1* *C*
8,9‐Dihydroxyeicosatrienoic acid	8,9‐DiHETrE	0.3516	0.0720 to 0.6313	0.0605*	*Figure* *1* *D*
15‐Deoxy‐delta 12,14‐prostaglandin J2	metPGJ2	0.4644	0.0705 to 0.8582	0.0724*	*Figure* *1* *E*
(+/−)‐20‐Hydroxy‐4Z,7Z,10Z,13Z,16Z,18E‐docosahexaenoic acid	20‐HDoHE	0.3988	0.0291 to 0.7686	0.0986*	*Figure* *1* *F*
14,15‐Dihydroxyeicosatrienoic acid	14,15‐DiHETrE	0.3408	−0.0365 to 0.7180	0.1850	*Figure* *S1* *A*
11,12‐Dihydroxyeicosatrienoic acid	11,12‐DiHETrE	0.2325	−0.0961 to 0.5610	0.3475	*Figure* *S1* *B*
15(S)‐Hydroxyeicosatetraenoic acid	15(S)‐HETE	0.2085	−0.1234 to 0.5403	0.4074	*Figure* *S1* *C*
Leukotriene C4	LTC4	0.2551	−0.2070 to 0.7171	0.4694	*Figure* *S1* *D*
13‐keto‐9Z,11E‐Octadecadienoic acid	13‐Oxo‐ODE	0.1743	−0.2191 to 0.5676	0.5609	*Figure* *S1* *E*
9‐Hydroxyeicosatetraenoic acid	9‐HETE	0.0969	−0.1286 to 0.3224	0.5609	*Figure* *S1* *F*
20‐Hydroxyeicosatetraenoic acid	20‐HETE	0.1350	−0.2147 to 0.4848	0.5828	*Figure* *S1* *G*
(+/−)‐10‐Hydroxy‐4Z,7Z,11E,13Z,16Z,19Z‐docosahexaenoic acid	10‐HDoHE	−0.1061	−0.4414 to 0.2293	0.6124	*Figure* *S1* *H*
5,6‐Dihydroxy‐8Z,11Z,14Z‐eicosatrienoic acid	5,6‐DiHETrE	0.1062	−0.2366 to 0.4489	0.6124	*Figure* *S1* *I*
5(S)‐Hydroxyeicosatetraenoic acid	5(S)‐HETE	0.0830	−0.3082 to 0.4741	0.7170	*Figure* *S1* *J*
12‐epi‐Leukotriene B4	12‐epi‐LTB4	0.0325	−0.2466 to 0.3116	0.8178	*Figure* *S1* *K*

CI, confidence interval; FC, fold change; FDR, false discovery rate.

* FDRs < 0.10 were considered statistically significant.

### The eicosanoid 8,9‐DiHETrE independently predicts mortality in functional mitral regurgitation patients

A total of 22 patients (27.5%) died after a median follow‐up of 43 [38, 48] months. Of those, 14 patients (63.3%) had severe FMR. In patients with severe FMR, ROC analysis revealed an 8,9‐DiHETrE log_2_ value of −6.05 to be an optimal cut‐off to predict all‐cause mortality, with a Youden index of 0.511, a sensitivity of 57.1%, and a specificity of 93.9% [ROC area under the curve (AUC) 0.753 (95% CI 0.583–0.923), *P* = 0.003]. This observation did not hold true for the other five eicosanoids increased in severe FMR (11,12‐EET, 13(R)‐HODE, 12(S)‐HETE, metPGJ2, and 20‐HDoHE; data not shown). Thus, further outcome analyses were confined to 8,9‐DiHETrE.

**Figure 3:**
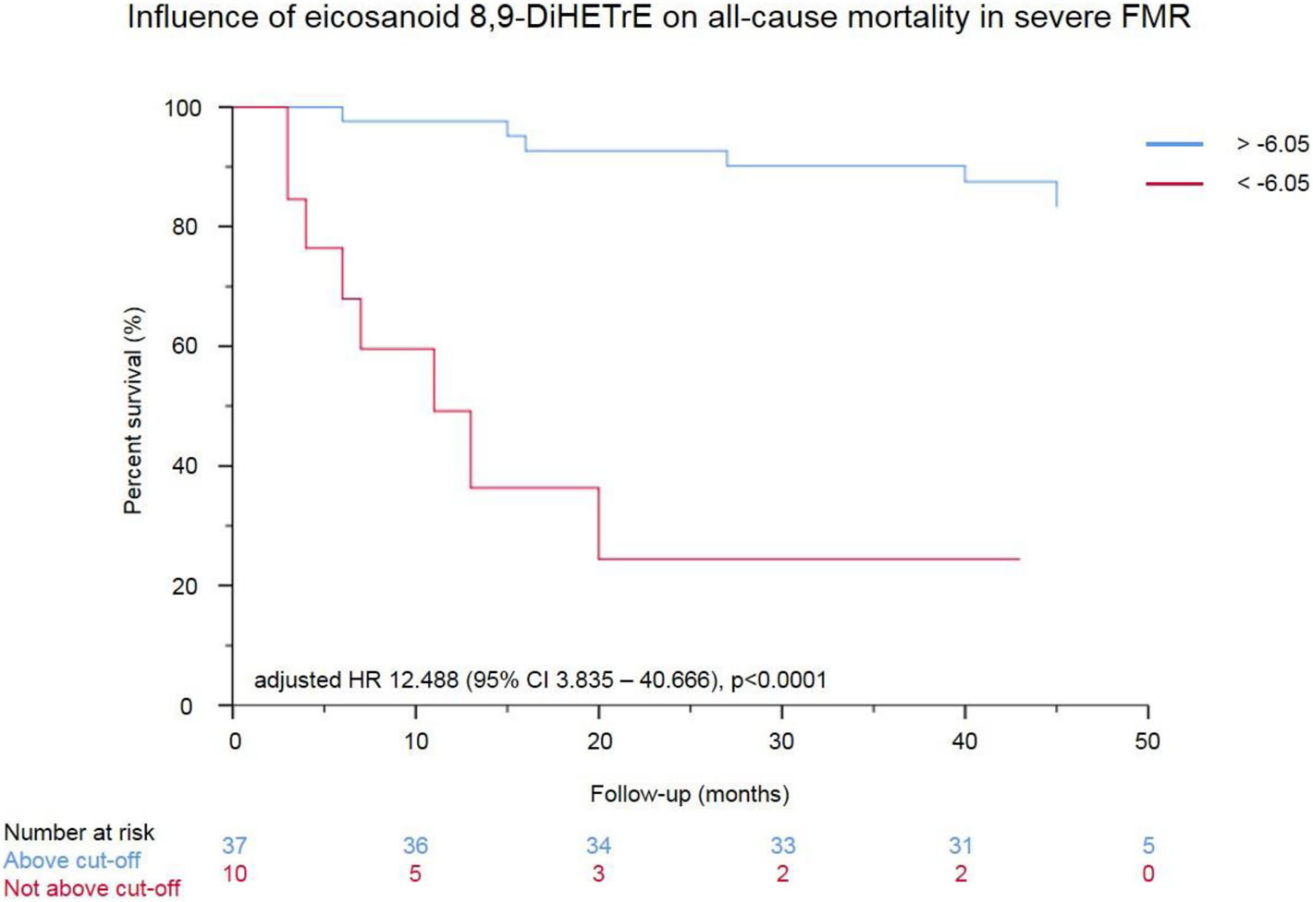
Influence of 8,9‐DiHETrE on outcome in patients with severe functional mitral regurgitation (FMR). All‐cause mortality of patients suffering from severe FMR. The Kaplan–Meier curves stratify by levels of 8,9‐DiHETrE. A cut‐off of −6.05 was identified using receiver‐operating characteristic (ROC) analysis. Censored patients are not shown. Multivariable Cox regression, alpha‐level 0.05. CI, confidence interval; HR, hazard ratio.

Cox regression analysis was employed to assess the influence of eicosanoids on outcome (*Table* *3*). Only eicosanoids that were significantly higher in severe FMR compared with patients with no or mild FMR were analysed. In a univariate analysis, 8,9‐DiHETrE predicted all‐cause mortality [crude HR 2.450 (95% CI 1.319–4.969), *P* = 0.005]. After multivariable adjustment for age and NT‐proBNP levels (variables observed to be associated with outcome in univariate analyses), 8,9‐DiHETrE remained predictive in patients with severe FMR [HR 3.754 (95% CI 1.739–8.102), *P* = 0.001], but not in patients with no or mild FMR [HR 0.286 (95% CI 0.032–2.541), *P* = 0.261]. When the cut‐off for 8,9‐DiHETrE determined by ROC analysis was applied, adjusted HR in severe FMR patients was 12.488 (95% CI 3.835–40.666; *P* < 0.0001). A respective Kaplan–Meier curve is provided in *Figure* *3*.

**Table 3:** Influence of eicosanoids on all‐cause mortality in severe functional mitral regurgitation

	Univariable			Multivariable—all patients			Multivariable—no/mild FMR			Multivariable—severe FMR		
Factor	HR	95% CI	*P*‐value	Adj. HR	95% CI	*P*‐value	Adj. HR	95% CI	*P*‐value	Adj. HR	95% CI	*P*‐value
Age	1.041	1.006–1.077	0.023*	1.028	0.992–1.065	0.129	1.036	0.967–1.109	0.316	1.027	0.981–1.074	0.252
Male sex	0.585	0.173–1.976	0.388									
Ischaemic HFrEF	1.799	0.779–4.154	0.169									
Diabetes mellitus	1.703	0.727–3.989	0.220									
BMI	1.007	0.998–1.016	0.117									
Arterial hypertension	1.996	0.736–5.412	0.175									
Hyperlipidaemia	1.526	0.651–3.576	0.331									
Atrial fibrillation	1.786	0.774–4.122	0.174									
ICD	0.629	0.256–1.544	0.312									
CRT	1.492	0.550–4.045	0.432									
Pacemaker	1.482	0.346–6.349	0.596									
NT‐proBNP per SD	1.380	1.053–1.808	0.020*	1.245	0.928–1.671	0.144	1.051	0.257–4.302	0.944	1.282	0.927–1.733	0.133
LVEF	1.034	0.969–1.103	0.315									
MV VCW	1.052	0.973–1.137	0.201									
11,12‐EET	1.820	0.798–4.151	0.154									
13(R)‐HODE	2.950	0.589–14.777	0.188									
12(S)‐HETE	1.020	0.643–1.619	0.933									
8,9‐DiHETrE	2.975	1.612–5.489	<0.001*	2.560	1.319–4.969	0.005*	0.286	0.032–2.541	0.261	3.754	1.739–8.102	0.001*
metPGJ2	1.520	0.969–2.384	0.068									
20‐HDoHE	0.931	0.541–1.603	0.797									

BMI, body mass index; CI, confidence interval; CRT, cardiac resynchronization therapy; FMR, functional mitral regurgitation; HFrEF, heart failure with reduced ejection fraction; HR, hazard ratio; ICD, implantable cardiac defibrillator; LVEF, left ventricular ejection fraction; MV, mitral valve; NT‐proBNP, N‐terminal pro‐brain natriuretic peptide; SD, standard deviation; VCW, vena contracta width.

Cox regression analysis was used to assess the influence of eicosanoids on all‐cause mortality in patients with severe or no functional mitral regurgitation, after a median follow‐up of 43 [38, 48] months.

* *P*‐values < 0.05 were considered statistically significant.

## Discussion

In this study, we employed metabolomic profiling in patients suffering from severe FMR. Upon multiple‐testing corrected statistical analysis, we found no significant metabolite regulations but observed up‐regulation of six eicosanoids in severe FMR when compared with patients with no or mild FMR. 8,9‐DiHETrE independently predicted all‐cause mortality in severe FMR.

Until recently, treatment of FMR was focused on management of the underlying cause leading to MR. However, mounting evidence implies that FMR cannot be considered a mere bystander or surrogate of disease; rather, FMR emerges as a separate clinical entity. Thus, investigating the pathomechanisms of FMR is imperative to provide treatment options before closure of the putative therapeutic window, after which no improvement in prognosis by intervention is possible. In this study, we specifically compared patients with severe FMR (i.e. patients where intervention is not expected to result in significant clinical improvement) with patients without or with only mild FMR. Metabolic alterations in the former cohort might provide important insights into why severe FMR in these patients is beyond repair.

The notion of a therapeutic window of opportunity, during which treatment of FMR might improve prognosis, is supported on a mechanistic level by an animal model investigating sheep subjected to apical myocardial infarction (not *per se* inducing FMR) and subsequent implantation of a graft‐based ventriculo‐atrial shunt (mimicking FMR). Timely removal of this shunt reversed LV remodelling.^22^ However, when the shunt was removed at a later time point, these adverse effects could not be remedied.^23^ This coincided with sustained loss of pro‐hypertrophic and anti‐apoptotic mediators in myocardial biopsy specimens.

We recently showed that certain microRNAs, which regulate expression of messenger RNAs,^24^ are decreased in HFrEF patients with severe FMR compared with patients without FMR, with increased microRNA‐133a being associated with poor prognosis.^17^ Apart from microRNA analysis, which depends on selection of a low number of targets, metabolomics in general and lipidomics specifically are increasingly employed, highlighting that various bioactive lipids may modulate cardiovascular disease.^25^

Eicosanoids are an important group of lipids involved in signalling and a wide array of physiological pathways on a systemic and local scale.^12^, ^26^ Eicosanoids are derived from arachidonic acid and synthesized either non‐enzymatically or via cyclooxygenase and lipoxygenase of the cytochrome P450 system.^27^, ^28^ In mice with advanced HF, increased activity of the cyclooxygenase pathway was observed, along with increased synthesis of 15‐HETE.^29^ One study observed that mitochondria isolated from patients suffering from HF exhibited aberrant eicosanoid synthesis patterns, leading to excess formation of pro‐inflammatory, pro‐apoptotic, and pro‐necrotic eicosanoids.^30^ Until now, the role of eicosanoids in FMR, which frequently complicates HF in humans, remained to be investigated.

Targeting a total of 17 eicosanoids, we found six to be increased in severe FMR: 11,12‐EET, 13(R)‐HODE, 12(S)‐HETE, 8,9‐DiHETrE, metPGJ2, and 20‐HDoHE. 8,9‐DiHETrE is synthesized from arachidonic acid via the cytochrome P450 system,^31^, ^32^, ^33^ indicating an increased activity of this metabolic pathway in severe FMR. We found that 8,9‐DiHETrE independently predicted all‐cause mortality in patients with, but not in patients without, severe FMR. A trial of patients with peripheral artery disease demonstrated 8,9‐DiHETrE to be associated with cardiovascular events, including acute coronary syndrome with an odds ratio of 92.^34^ However, in patients with primary prevention implantable cardiac defibrillator (ICD), 8,9‐DiHETrE was not associated with mortality; yet, levels were associated with a decreased risk of appropriate shocks.^35^ Of note, the frequency of severe FMR was not reported in this trial.

The causes of increased eicosanoids in severe FMR are unclear. Ibuprofen was shown to increase plasma levels of 8,9‐DiHETrE.^32^ In our cohort, differences in eicosanoids between patient groups were independent of background therapy, including acetylsalicylic acid (no patient received ibuprofen or diclofenac), thus ruling out pharmacological confounding of results. Another cause of eicosanoid increase might be inflammatory signalling. Subsequent studies into levels of pro‐inflammatory cytokines including interleukin‐6 or tumour necrosis factor‐alpha in relation to expression of eicosanoids are required to answer this hypothesis.

The consequences of eicosanoid derangement in severe FMR remain equally elusive. In mice, 8,9‐DiHETrE was shown to induce vasodilation, decreasing blood pressure.^36^ We were unable to corroborate this in humans, as 8,9‐DiHETrE did not correlate with blood pressure (data not shown). However, the fact that 8,9‐DiHETrE independently predicted mortality indicates that the eicosanoid is mechanistically involved in progression of HF. Correspondingly, NT‐proBNP and 8,9‐DiHETrE were positively correlated in severe FMR patients, but not in patients without severe FMR. Yet, NT‐proBNP was similar between these two patient groups. We observed a correlation between 8,9‐DiHETrE and MV VCW as a marker of MR severity. Intriguingly, this correlation did not exist in patients with severe FMR only, which indicates that 8,9‐DiHETrE increase is not a consequence of progressive haemodynamic deterioration but of the presence of severe FMR *per se*. 8,9‐DiHETrE might thus influence prognosis independently of volume overload and functional deterioration, alluding to pathomechanisms not uncovered by our study.

This study has important limitations. Data presented here are observational. Hence, causality cannot be inferred. Rather, our observations might serve as a nucleus for further investigations, assessing the role of eicosanoids in the pathogenesis of HF in general and FMR specifically, both *in vitro* and in patients. Sample size (*n* = 80) was relatively small; larger studies in an external, validation cohort might enable more detailed analyses regarding eicosanoid levels and outcome. Given these uncertainties, therapeutic application of eicosanoids in general, or 8,9‐DiHETrE specifically, cannot be insinuated. In our cohort, there was a large preponderance of male compared with female patients; as such, an influence of sex on eicosanoids cannot be assessed. However, the prevalence of male sex in our study was similar to other clinical trials investigating HF patients.^37^, ^38^, ^39^

In conclusion, by using a metabolomic approach, we demonstrate the up‐regulation of various eicosanoids in severe FMR. Among these assessed eicosanoids, 8,9‐DiHETrE may be associated with poor prognosis. Severe FMR appears to entail crucial derangements in eicosanoid metabolism, driving disease progression. These changes in turn might influence prognosis. Further studies are required to assess the mechanistic impact of these eicosanoids on FMR, potentially opening new therapeutic targets for intervention.

## Conflict of interest

The authors have no conflicts of interest to declare.

## Funding

This study was supported by the Austrian Science Fund (FWF, KLI‐818B).

## Supporting information


**Figure S1.** Normalization of eicosanoid measurements.
Normalization of eicosanoids was performed using the pairwise cyclic loess method as implemented in R‐package limma. A) non‐normalized data; B) normalized data. Metabolite values were not normalized.
**Figure S2.** Levels of eicosanoids in patients with severe vs. no or mild functional mitral regurgitation.
Eicosanoids were measured in plasma from patients with or without severe FMR using metabolomic analysis. A) 14,15‐DiHETrE, B) 11,12‐DiHETrE, C) 15(S)‐HETE, D) LTC4, E) 13‐Oxo‐ODE, F) 9‐HETE, G) 20‐HETE, H) 10‐HDoHE, I) 5,6‐DiHETrE, J) 5(S)‐HETE, K) 12‐epi‐LTB4. FDR false discovery rate, FMR functional mitral regurgitation. FDR level 0.1.
**Table S1.** Metabolites in severe functional mitral regurgitation.
