# Supplementary material for: Metabolomics implicate eicosanoids in severe functional mitral regurgitation
Source: ESC Heart Fail. 2022 Oct 10;10(1):311–21. doi: 10.1002/ehf2.14160 (PMC9871691; doi:10.1002/ehf2.14160)
Supplement: Supplementary file 1 — Figure S1. Normalization of eicosanoid measurements. Normalization of eicosanoids was performed using the pairwise cyclic loess method as implemented in R‐package limma. A) non‐normalized data; B) normalized data. Metabolite values were not normalized. Figure S2. Levels of eicosanoids in patients with severe vs. no or mild functional mitral regurgitation. Eicosanoids were measured in plasma from patients with or without severe FMR using metabolomic analysis. A) 14,15‐DiHETrE, B) 11,12‐DiHETrE, C) 15(S)‐HETE, D) LTC4, E) 13‐Oxo‐ODE, F) 9‐HETE, G) 20‐HETE, H) 10‐HDoHE, I) 5,6‐DiHETrE, J) 5(S)‐HETE, K) 12‐epi‐LTB4. FDR false discovery rate, FMR functional mitral regurgitation. FDR level 0.1. Table S1. Metabolites in severe functional mitral regurgitation. [file EHF2-10-311-s001.docx]

## Supplemental Figure S1. Normalization of eicosanoid measurements.


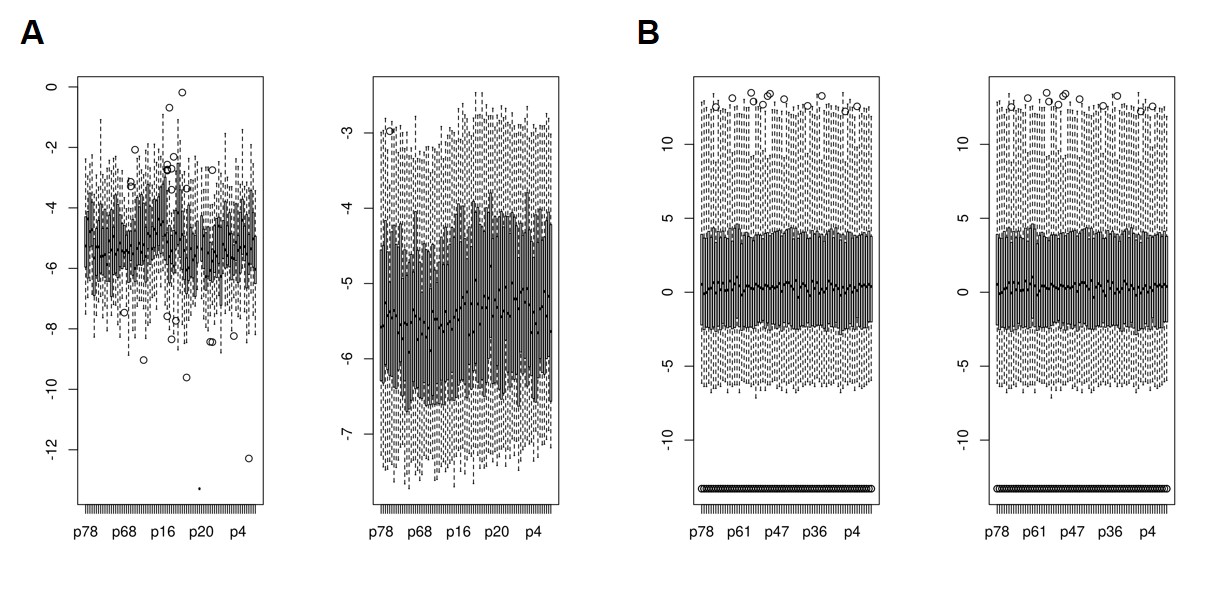


Normalization of eicosanoids was performed using the pairwise cyclic loess method as implemented in R-package limma. A) non-normalized data; B) normalized data. Metabolite values were not normalized

## Supplemental Figure S2. Levels of eicosanoids in patients with severe vs. no or mild functional mitral regurgitation.


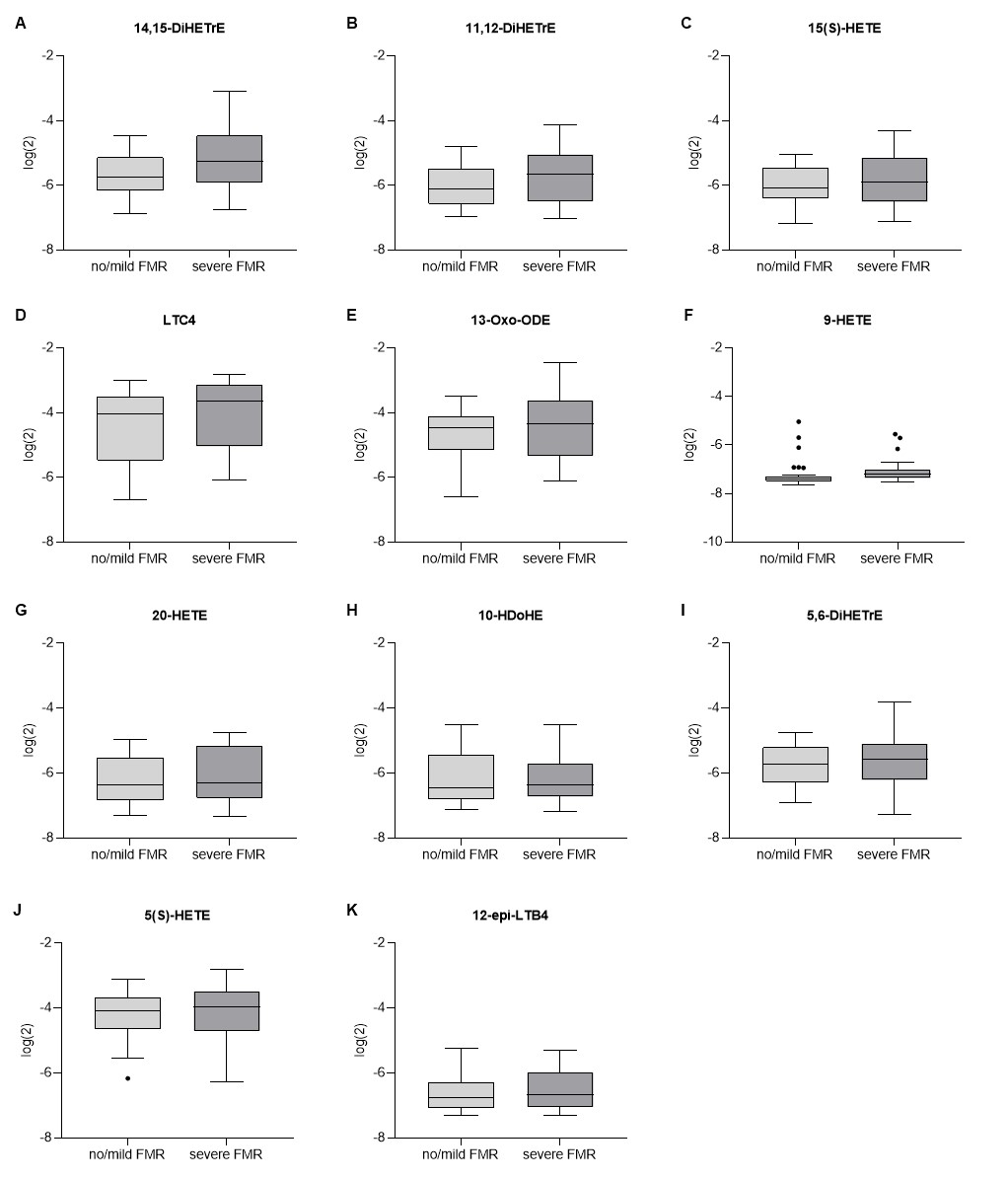


Eicosanoids were measured in plasma from patients with or without severe FMR using metabolomic analysis. A) 14,15-DiHETrE, B) 11,12-DiHETrE, C) 15(S)-HETE, D) LTC4, E) 13-Oxo-ODE, F) 9-HETE, G) 20-HETE, H) 10-HDoHE, I) 5,6-DiHETrE, J) 5(S)-HETE, K) 12-epi-LTB4. FDR false discovery rate, FMR functional mitral regurgitation. FDR level 0.1

## Supplemental Table S1. Metabolites in severe functional mitral regurgitation

| **Metabolite ID** | **Log_2_ FC** | **95% CI** | **FDR** |
| --- | --- | --- | --- |
|  | 0.1243 | -0.1809 – 0.4294 | 0.9999 |
| **Acrylcarnitines** |  |  |  |
| Carnitine | 0.2158 | -0.0455 - 0.4772 | 0.9999 |
| Acetylcarnitine | 0.2109 | -0.0922 - 0.5140 | 0.9999 |
| Propionylcarnitine | 0.1345 | -0.1669 - 0.4359 | 0.9999 |
| Propenoylcarnitine | 0.0876 | -0.0589 - 0.2341 | 0.9999 |
| Hydroxypropionylcarnitine | 0.0685 | -0.1168 - 0.2537 | 0.9999 |
| Butyrylcarnitine | -0.1385 | -0.3991 - 0.1221 | 0.9999 |
| Butenylcarnitine | 0.0948 | -0.0874 - 0.2771 | 0.9999 |
| Hydroxybutyrylcarnitine | 0.0218 | -0.2510 - 0.2947 | 1.0000 |
| Valerylcarnitine | 0.0673 | -0.1787 - 0.3132 | 0.9999 |
| Tiglylcarnitine | 0.0629 | -0.0823 - 0.2081 | 0.9999 |
| Glutaconylcarnitine | 0.0413 | -0.0797 - 0.1624 | 0.9999 |
| Glutarylcarnitine (Hydroxyhexanoylcarnitine) | 0.1374 | -0.0431 - 0.3180 | 0.9999 |
| Methylglutarylcarnitine | -0.0149 | -0.1524 - 0.1225 | 1.0000 |
| Hydroxyvalerylcarnitine (Methylmalonylcarnitine) | 0.0557 | -0.0963 - 0.2076 | 0.9999 |
| Hexanoylcarnitine (Fumarylcarnitine) | 0.0489 | -0.0785 - 0.1763 | 0.9999 |
| Hexenoylcarnitine | 0.0450 | -0.1098 - 0.1998 | 0.9999 |
| Pimelylcarnitine | 0.1884 | -0.0323 - 0.4091 | 0.9999 |
| Octanoylcarnitine | -0.0111 | -0.2552 - 0.2331 | 1.0000 |
| Nonaylcarnitine | 0.1650 | -0.0329 - 0.3630 | 0.9999 |
| Decanoylcarnitine | 0.0080 | -0.2141 - 0.2302 | 1.0000 |
| Decenoylcarnitine | 0.0406 | -0.1653 - 0.2466 | 0.9999 |
| Decadienylcarnitine | 0.0521 | -0.1531 - 0.2573 | 0.9999 |
| Dodecanoylcarnitine | 0.0696 | -0.1546 - 0.2938 | 0.9999 |
| Dodecenoylcarnitine | 0.0356 | -0.2080 - 0.2792 | 1.0000 |
| Dodecanedioylcarnitine | -0.0024 | -0.0680 - 0.0363 | 1.0000 |
| Tetradecanoylcarnitine | 0.0663 | -0.1507 - 0.2834 | 0.9999 |
| Tetradecenoylcarnitine | 0.1403 | -0.1290 - 0.4097 | 0.9999 |
| Hydroxytetradecenoylcarnitine | 0.0856 | -0.0996 - 0.2707 | 0.9999 |
| Tetradecadienylcarnitine | 0.1262 | -0.2131 - 0.4654 | 0.9999 |
| Hydroxytetradecadienylcarnitine | 0.1357 | 0.0023 - 0.2691 | 0.9999 |
| Hexadecanoylcarnitine | 0.0141 | -0.2024 - 0.2305 | 1.0000 |
| Hexadecenoylcarnitine | 0.0491 | -0.1484 - 0.2466 | 0.9999 |
| Hydroxyhexadecenoylcarnitine | 0.0095 | -0.1774 - 0.1964 | 1.0000 |
| Hexadecadienylcarnitine | 0.1442 | -0.0793 - 0.3676 | 0.9999 |
| Hydroxyhexadecadienylcarnitine | 0.0443 | -0.0894 - 0.1779 | 0.9999 |
| Hydroxyhexadecanoylcarnitine | 0.0790 | -0.0342 - 0.1922 | 0.9999 |
| Octadecanoylcarnitine | 0.0712 | -0.1948 - 0.3372 | 0.9999 |
| Octadecenoylcarnitine | 0.0931 | -0.1769 - 0.3631 | 0.9999 |
| Hydroxyoctadecenoylcarnitine | 0.0349 | -0.1098 - 0.7970 | 0.9999 |
| Octadecadienylcarnitine | 0.0351 | -0.2572 - 0.3274 | 1.0000 |
|  |  |  |  |
| **Amino Acids and Biogenic Amines** |  |  |  |
| Alanine | -0.1062 | -0.2824 - 0.0701 | 0.9999 |
| Arginine | -0.1519 | -0.4395 - 0.1357 | 0.9999 |
| Asparagine | -0.0355 | -0.1909 - 0.1200 | 0.9999 |
| Aspartate | -0.0880 | -0.2960 - 0.1200 | 0.9999 |
| Citrulline | 0.0698 | -0.2588 - 0.3983 | 0.9999 |
| Glutamine | 0.0238 | -0.0953 - 0.1429 | 0.9999 |
| Glutamate | -0.2389 | -0.5052 - 0.0275 | 0.9999 |
| Glycine | -0.0826 | -0.2590 - 0.0939 | 0.9999 |
| Histidine | -0.0199 | -0.1421 - 0.1024 | 1.0000 |
| Isoleucine | -0.0503 | -0.2161 - 0.1156 | 0.9999 |
| Leucine | -0.1195 | -0.3068 - 0.0678 | 0.9999 |
| Lysine | -0.0893 | -0.2182 - 0.0396 | 0.9999 |
| Methionine | 0.0650 | -0.1141 - 0.2441 | 0.9999 |
| Ornithine | -0.0194 | -0.2176 - 0.1787 | 1.0000 |
| Phenylalanine | 0.0807 | -0.0735 - 0.2349 | 0.9999 |
| Proline | -0.2638 | -0.4954 - -0.0322 | 0.9999 |
| Serine | 0.0086 | -0.1513 - 0.1684 | 1.0000 |
| Threonine | -0.0148 | -0.2296 - 0.1999 | 1.0000 |
| Tryptophan | 0.0561 | -0.1492 - 0.2613 | 0.9999 |
| Tyrosine | 0.0246 | -0.1709 - 0.2202 | 1.0000 |
| Valine | -0.0594 | -0.2324 - 0.1136 | 0.9999 |
| Acetylornithine | -0.0029 | -0.4849 - 0.4792 | 1.0000 |
| Asymmetric dimethylarginine | -0.0856 | -0.2436 - 0.0724 | 0.9999 |
| Symmetric dimethylarginine | -0.8158 | -2.4882 - 0.8566 | 0.9999 |
| alpha-Aminoadipic acid | -0.2924 | -0.6102 - 0.0253 | 0.9999 |
| Carnosine | 1.1244 | -0.3302 - 2.5790 | 0.9999 |
| Creatinine | 0.0297 | -0.2416 - 0.3009 | 1.0000 |
| Histamine | -0.0065 | -0.0433 - 0.0302 | 1.0000 |
| Kynurenine | -0.0220 | -0.2905 - 0.2466 | 1.0000 |
| Methioninesulfoxide | -0.0550 | -0.3363 - 0.2262 | 0.9999 |
| Nitrotyrosine | 0.2941 | -1.100 - 1.6881 | 0.9999 |
| cis-4-Hydroxyproline | 0.0000 | -0.0022 - 0.0022 | 1.0000 |
| trans-OH-Pro | -0.1100 | -0.3804 - 0.1604 | 0.9999 |
| Phenylethylamine | 0.0000 | -0.0022 - 0.0022 | 1.0000 |
| Putrescine | -0.0435 | -0.2982 - 0.2112 | 1.0000 |
| Serotonin | -0.8353 | -2.1890 - 0.5184 | 0.9999 |
| Spermidine | 0.0015 | -0.2209 - 0.2239 | 1.0000 |
| Spermine | 0.0200 | -0.0622 - 0.1021 | 0.9999 |
| Taurine | 0.0421 | -0.1740 - 0.2583 | 0.9999 |
| Dopamine | 1.7902 | -0.7904 - 4.3709 | 0.9999 |
| DOPA | 0.0353 | -2.5488 - 2.6194 | 1.0000 |
|  |  |  |  |
| **Monosaccharides** |  |  |  |
| Hexose | -0.0721 | -0.2640 - 0.1198 | 0.9999 |
|  |  |  |  |
| **Sphingolipids** |  |  |  |
| SM(OH) C14:1 | 0.1569 | -0.0822 - 0.3960 | 0.9999 |
| SM C16:0 | 0.1118 | -0.0531 - 0.2766 | 0.9999 |
| SM C16:1 | 0.1379 | -0.0492 - 0.3249 | 0.9999 |
| SM (OH) C16:1 | 0.1671 | -0.0602 - 0.3945 | 0.9999 |
| SM C18:0 | 0.1390 | -0.0927 - 0.3708 | 0.9999 |
| SM C18:1 | 0.1696 | -0.0722 - 0.4114 | 0.9999 |
| SM C20:2 | 0.1845 | -0.0310 - 0.4000 | 0.9999 |
| SM C22:3 | 0.4820 | -0.3827 - 1.3468 | 0.9999 |
| SM (OH) C22:1 | 0.1280 | -0.1054 - 0.3614 | 0.9999 |
| SM (OH) C22:2 | 0.1551 | -0.0608 - 0.3711 | 0.9999 |
| SM C24:0 | 0.0190 | -0.1780 - 0.2161 | 1.0000 |
| SM C24:1 | 0.1486 | -0.0367 - 0.3339 | 0.9999 |
| SM (OH) C24:1 | 0.0275 | -0.1776 - 0.2325 | 1.0000 |
| SM C26:0 | 0.0560 | -0.1559 - 0.2678 | 0.9999 |
| SM C26:1 | 0.0290 | -0.1773 - 0.2353 | 1.0000 |
|  |  |  |  |
| **Glycerophospholipids** |  |  |  |
| lysoPC a C14:0 | -0.0508 | -0.1497 - 0.0481 | 0.9999 |
| lysoPC a C16:0 | -0.0309 | -0.2170 - 0.1552 | 1.0000 |
| lysoPC a C16:1 | -0.1734 | -0.4321 - 0.0853 | 0.9999 |
| lysoPC a C17:0 | 0.0206 | -0.2100 - 0.2512 | 1.0000 |
| lysoPC a C18:0 | -0.0331 | -0.2304 - 0.1643 | 1.0000 |
| lysoPC a C18:1 | -0.0476 | -0.2478 - 0.1527 | 0.9999 |
| lysoPC a C18:2 | 0.0192 | -0.2397 - 0.2781 | 1.0000 |
| lysoPC a C20:3 | -0.2042 | -0.4475 - 0.0390 | 0.9999 |
| lysoPC a C20:4 | -0.0524 | -0.2972 - 0.1924 | 0.9999 |
| lysoPC a C24:0 | -0.1677 | -0.3955 - 0.0600 | 0.9999 |
| lysoPC a C26:0 | -0.2691 | -0.5658 - 0.0276 | 0.9999 |
| lysoPC a C26:1 | -0.2311 | -0.4995 - 0.0373 | 0.9999 |
| lysoPC a C28:0 | -0.1282 | -0.3838 - 0.1274 | 0.9999 |
| lysoPC a C28:1 | -0.1328 | -0.3716 - 0.1060 | 0.9999 |
| PC aa C24:0 | -0.2445 | -0.5367 - 0.0477 | 0.9999 |
| PC aa C26:0 | -0.1617 | -0.3884 - 0.0650 | 0.9999 |
| PC aa C28:1 | 0.0481 | -0.1933 - 0.2894 | 0.9999 |
| PC aa C30:0 | -0.0243 | -0.2797 - 0.2311 | 1.0000 |
| PC aa C30:2 | 0.2263 | -0.0434 - 0.4960 | 0.9999 |
| PC aa C32:0 | 0.0307 | -0.1288 - 0.1903 | 0.9999 |
| PC aa C32:1 | -0.1833 | -0.5397 - 0.1731 | 0.9999 |
| PC aa C32:2 | -0.1722 | -0.5582 - 0.2138 | 0.9999 |
| PC aa C32:3 | 0.0877 | -0.1495 - 0.3250 | 0.9999 |
| PC aa C34:1 | -0.0345 | -0.2138 - 0.1448 | 0.9999 |
| PC aa C34:2 | 0.0791 | -0.0672 - 0.2255 | 0.9999 |
| PC aa C34:3 | -0.0592 | -0.3274 - 0.2090 | 0.9999 |
| PC aa C34:4 | -0.1529 | -0.5162 - 0.2103 | 0.9999 |
| PC aa C36:0 | 0.0012 | -0.2227 - 0.2251 | 1.0000 |
| PC aa C36:1 | -0.0934 | -0.2995 - 0.1126 | 0.9999 |
| PC aa C36:2 | 0.0333 | -0.1280 - 0.1946 | 0.9999 |
| PC aa C36:3 | -0.0469 | -0.2293 - 0.1356 | 0.9999 |
| PC aa C36:4 | 0.0015 | -0.1925 - 0.1954 | 1.0000 |
| PC aa C36:5 | -0.2876 | -0.6975 - 0.1223 | 0.9999 |
| PC aa C36:6 | -0.1610 | -0.5303 - 0.2083 | 0.9999 |
| PC aa C38:0 | -0.0031 | -0.2401 - 0.2339 | 1.0000 |
| PC aa C38:1 | -0.0308 | -0.2792 - 0.2176 | 1.0000 |
| PC aa C38:3 | -0.1173 | -0.3179 - 0.0833 | 0.9999 |
| PC aa C38:4 | -0.0211 | -0.2125 - 0.1703 | 1.0000 |
| PC aa C38:5 | -0.0961 | -0.2983 - 0.1061 | 0.9999 |
| PC aa C38:6 | -0.0033 | -0.2574 - 0.2509 | 1.0000 |
| PC aa C40:1 | -0.0485 | -0.2109 - 0.1139 | 0.9999 |
| PC aa C40:2 | -0.0206 | -0.2294 - 0.1882 | 1.0000 |
| PC aa C40:3 | -0.0650 | -0.2529 - 0.1229 | 0.9999 |
| PC aa C40:4 | -0.0593 | -0.2689 - 0.1502 | 0.9999 |
| PC aa C40:5 | -0.1156 | -0.3513 - 0.1202 | 0.9999 |
| PC aa C40:6 | -0.0819 | -0.3419 - 0.1781 | 0.9999 |
| PC aa C42:0 | 0.0859 | -0.1590 - 0.3308 | 0.9999 |
| PC aa C42:1 | 0.0122 | -0.2156 - 0.2400 | 1.0000 |
| PC aa C42:2 | -0.0690 | -0.2454 - 0.1073 | 0.9999 |
| PC aa C42:4 | -0.0128 | -0.1691 - 0.1434 | 1.0000 |
| PC aa C42:5 | -0.0683 | -0.3034 - 0.1668 | 0.9999 |
| PC aa C42:6 | -0.1353 | -0.3335 - 0.0628 | 0.9999 |
| PC ae C30:0 | -0.0196 | -0.2333 - 0.1940 | 1.0000 |
| PC ae C30:1 | -0.0643 | -0.3090 - 0.1804 | 0.9999 |
| PC ae C30:2 | -0.0070 | -0.2175 - 0.2036 | 1.0000 |
| PC ae C32:1 | 0.0561 | -0.1396 - 0.2518 | 0.9999 |
| PC ae C32:2 | 0.0022 | -0.1913 - 0.1957 | 1.0000 |
| PC ae C34:0 | 0.0345 | -0.1652 - 0.2342 | 1.0000 |
| PC ae C34:1 | 0.0217 | -0.1537 - 0.1972 | 1.0000 |
| PC ae C34:2 | 0.1163 | -0.1001 - 0.3328 | 0.9999 |
| PC ae C34:3 | 0.0640 | -0.1846 - 0.3125 | 0.9999 |
| PC ae C36:0 | 0.0135 | -0.1737 - 0.2007 | 1.0000 |
| PC ae C36:1 | 0.0161 | -0.1695 - 0.2018 | 1.0000 |
| PC ae C36:3 | 0.0425 | -0.1732 - 0.2582 | 0.9999 |
| PC ae C36:2 | 0.0946 | -0.1073 - 0.2964 | 0.9999 |
| PC ae C36:4 | 0.0916 | -0.1168 - 0.3000 | 0.9999 |
| PC ae C36:5 | 0.0362 | -0.1766 - 0.2489 | 1.0000 |
| PC ae C38:0 | -0.0896 | -0.3594 - 0.1801 | 0.9999 |
| PC ae C38:1 | -0.0687 | -0.2722 - 0.1347 | 0.9999 |
| PC ae C38:2 | 0.0029 | -0.1855 - 0.1913 | 1.0000 |
| PC ae C38:3 | -0.0057 | -0.2001 - 0.1886 | 1.0000 |
| PC ae C38:4 | 0.1129 | -0.0723 - 0.2982 | 0.9999 |
| PC ae C38:5 | 0.0460 | -0.1214 - 0.2135 | 0.9999 |
| PC ae C38:6 | -0.0069 | -0.2287 - 0.2149 | 1.0000 |
| PC ae C40:1 | 0.0037 | -0.2112 - 0.2187 | 1.0000 |
| PC ae C40:2 | 0.0304 | -0.1702 - 0.2311 | 1.0000 |
| PC ae C40:3 | 0.0384 | -0.1346 - 0.2113 | 0.9999 |
| PC ae C40:4 | 0.1379 | -0.0472 - 0.3229 | 0.9999 |
| PC ae C40:5 | 0.0863 | -0.0956 - 0.2682 | 0.9999 |
| PC ae C40:6 | 0.0361 | -0.1759 - 0.2481 | 1.0000 |
| PC ae C42:0 | -0.0625 | -0.1687 - 0.0438 | 0.9999 |
| PC ae C42:1 | -0.0101 | -0.1686 - 0.1484 | 1.0000 |
| PC ae C42:2 | -0.0291 | -0.2315 - 0.1733 | 1.0000 |
| PC ae C42:3 | 0.0694 | -0.1346 - 0.2733 | 0.9999 |
| PC ae C42:4 | 0.1314 | -0.1004 - 0.3631 | 0.9999 |
| PC ae C42:5 | 0.1140 | -0.0783 - 0.3062 | 0.9999 |
| PC ae C44:3 | -0.0622 | -0.2334 - 0.1089 | 0.9999 |
| PC ae C44:4 | 0.0983 | -0.1193 - 0.3159 | 0.9999 |
| PC ae C44:6 | 0.1482 | -0.0882 - 0.3845 | 0.9999 |
| PC ae C44:5 | 0.1750 | -0.0782 - 0.4282 | 0.9999 |
